# Adsorption Behavior of Surfactant on Lignite Surface: A Comparative Experimental and Molecular Dynamics Simulation Study

**DOI:** 10.3390/ijms19020437

**Published:** 2018-02-01

**Authors:** Meng He, Wei Zhang, Xiaoqiang Cao, Xiaofang You, Lin Li

**Affiliations:** College of Chemical and Environmental Engineering, Shandong University of Science and Technology, Qingdao 266590, China; hemeng2619@126.com (M.H.); zhangwei5736@163.com (W.Z.); xiaoqiangcao@126.com (X.C.); yxf19821212@163.com (X.Y.)

**Keywords:** molecular dynamics, XPS, adsorption, hydrophobicity, lignite, nonylphenol ethoxylate

## Abstract

Experimental and computational simulation methods are used to investigate the adsorption behavior of the surfactant nonylphenol ethoxylate (NPEO10), which contains 10 ethylene oxide groups, on the lignite surface. The adsorption of NPEO10 on lignite follow a Langmuir-type isotherm. The thermodynamic parameters of the adsorption process show that the whole process is spontaneous. X-ray photoelectron spectroscopic (XPS) analysis indicates that a significant fraction of the oxygen-containing functional groups on the lignitic surface were covered by NPEO10. Molecular dynamics (MD) simulations show that the NPEO10 molecules were found to adsorb at the water-coal interface. Moreover, polar interactions are the main effect in the adsorption process. The density distributions of coal, NPEO10, and water molecules along the Z axis show that the remaining hydrophobic portions of the surfactant extend into the solution, creating a more hydrophobic coal surface that repels water molecules. The negative interaction energy calculated from the density profiles of the head and tail groups along the three spatial directions between the surfactant and the lignitic surface suggest that the adsorption process is spontaneous. The self-diffusion coefficients show that the presence of NPEO10 causes higher water mobility by improving the hydrophobicity of lignite.

## 1. Introduction

Coal plays an important role in fulfilling the energy needs of our society. Lignite is a typical low-rank coal with very large deposits, which is easy to burn and has highly volatile components [1,2]. However, lignite has low heating values primarily because of its high oxygen and moisture contents, and it forms a great deal of coal slime in mining because lignite is weathered and fragmented easily. Drying and flotation are the main technologies used to improve the utilization ratio of lignite. Flotation is one of the most important methods to concentrate lignitic slimes, but low-rank coals, especially lignite, are generally known to be the most difficult coals to float [3,4,5,6,7]. Their low buoyancy has mainly been attributed to the high oxygen content and the abundance of hydrophilic functional groups at their surface [8]. Many researchers have tried to improve the hydrophobicity of difficult-to-float lignite by introducing appropriate agents. Kadim et al. [9] carried out a series of flotation tests with lignite from three mines. Vamvuka et al. [10] explained the electric charge condition on the surface of the particles of the lignite flotation process using the zeta potential. Yakup [11] proposed the use of a regime involving treatments with kerosene + emulsifier and kerosene + emulsifier + surfactant and conducted a flotation study. However, microscopic understanding is less frequently used in considerations of the adsorption of surfactants on the surface of coal. Zhang et al. [12] used sorbitan monooleate to pretreat lignite, enhancing the flotation of pretreated lignite. Xia et al. [13] found that a mixture of dodecane and 4-Dodecylphenol was an effective collector for lignite flotation. Ni et al. [14] found that the combustible matter recovery of lignite increased when the lignite was preconditioned with Tween 80 before addition to the collector.

In recent years, molecular dynamics simulation has become a valuable tool to investigate the interactions of water/surfactant (collector)/mineral surfaces. Compared with experimental methods, computer simulations can directly provide microscopic details and fundamental understanding [15]. Chen et al. [16] used the ReaxFF reactive force field for molecular dynamics simulations of the spontaneous combustion of lignite. Zhang [17] used molecular dynamics (MD) simulations to study the structure and dynamics of a brown coal matrix during the moisture removal process. Zhang et al. [18] investigated the wettability modification of Wender lignite by adsorption of dodecyl poly ethoxylated surfactants with different degrees of ethoxylation by molecular dynamics simulation. Rai et al. [19] studied the adsorption of oleate and dodecylammonium chloride molecules on spodumene and jadeite surfaces. Xu et al. [20] computed the interaction energies between water molecules/ammonium ions and the Muscovite (001) surface. Wang et al. [21] used MD simulations to describe the co-adsorption of a mixed surfactant (dodecylamine hydrochloride and sodium oleate) on the Muscovite surface in an aqueous solution. Although there have been several studies about molecular dynamics simulations on minerals, they have mainly focused on minerals of a single structural and chemical component. However, lignite is a matter system with structural complexity and chemical component diversity, which is mainly composed of 85–95% organic material. Therefore, the uniform chemical structure representing coal is nonexistent. Some assumptions must be made for investigating the coal structure. Lignite can be regarded as a highly cross-linked polymer consisting of lignite macromolecules through bridge bonds, hydrogen bonds, van der Waals force, and so on [22,23,24]. Therefore, few studies that illustrate the adsorption of surfactants on a lignite surface have been reported because of the structural complexity and chemical component diversity of lignite.

We investigated the fundamental properties and mechanism of the adsorption of nonylphenol ethoxylate (NPEO_10_), which contained 10 ethylene oxide groups, on the surface of lignite using MD and X-ray photoelectron spectroscopy (XPS). The adsorption of NPEO_10_ on the model surface of lignite was investigated using MD simulations. Quantifying the molecular-scale structural and dynamic behavior of the water/surfactant/coal system is helpful to improve understanding of the interactions between NPEO_10_ and lignite. In addition, adsorption experiments between lignite and NPEO_10_ were performed to verify the simulation results.

## 2. Results and Discussion

### 2.1. Adsorption Isotherms

**Figure 1:**
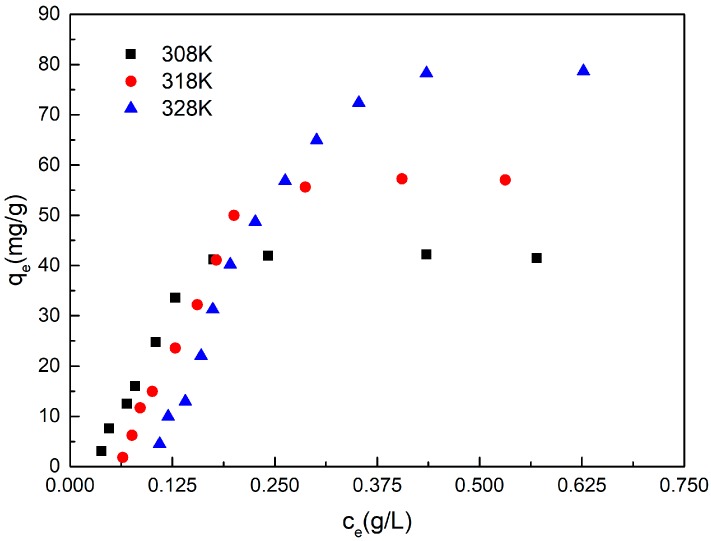
Adsorption isotherms of NPEO_10_ on lignite.

Adsorption isotherms of adsorptions of NPEO_10_ on the coal sample are illustrated in Figure 1 at 308, 318, and 328 K, respectively.

Figure 1 shows that the adsorption of NPEO_10_ on coal is sensitive to the effect of temperature. The linearized Langmuir Equation (1) and linearized Freundlich Equation (2) can be represented as follows [25,26,27,28]:

(1) $$C_{e}/q_{e}=1/bQ_{m}+C_{e}/Q_{m}$$

(2) $${\mathrm{lg}q}_{e}=\mathrm{lg}k+(1/n)\mathrm{lg}c_{e}$$

where *b* and *Q_m_* refer to the Langmuir constant, which is related to the affinity of the adsorption sites, and the maximum amount of NPEO_10_ per unit weight of adsorbent, and were calculated from the slope and intercept, respectively, of the straight lines of the plot *C_e_*/*q_e_* vs. *C_e_*. According to Equation (2), *k* and *n* refer to the Freundlich constant and can be determined from the linear plot of lg*q_e_* vs. *C_e_*. *k* is correlated to the adsorption capacity when the adsorbate equilibrium concentration of the adsorbate is equal to 1, and *n* represents the degree of dependence of the adsorption process on the equilibrium concentration.

The values of *Q_m_*, *b*, and *n* are summarized in Table 1. The isotherm data were calculated using the least squares method, and the related correlation coefficients (*r*^2^ values) are given in the same table. As shown in Table 1, the Langmuir equation represents the adsorption process very well; the *r*^2^ values of the Langmuir equation are 0.9990, 0.9996, and 0.9992 at 308, 318, and 328 K, respectively, all higher than the *r*^2^ values of the Freundlich equation, suggesting that the adsorption of NPEO_10_ onto the coal sample closely follows the Langmuir model.

The adsorption free energy (Δ*G*°) can be calculated using the following equation [29]:

(3) $$\Delta G^{o}=-RT\ln b$$

where *b*, *R*, and *T* are the Langmuir constant, ideal gas constant, and the absolute temperature, respectively. The adsorption free energy calculated on a molar basis was −44.80, −45.28, and −45.88 kJ/mol at 308, 318, and 328 K, respectively (as shown in Table 1). The value of Δ*G*° is negative, indicating a spontaneous process under the experimental conditions. The observed decrease in the Δ*G*° values with increasing temperature indicates that adsorption occurs more efficiently at higher temperatures.

### 2.2. XPS Analysis

**Figure 2:**
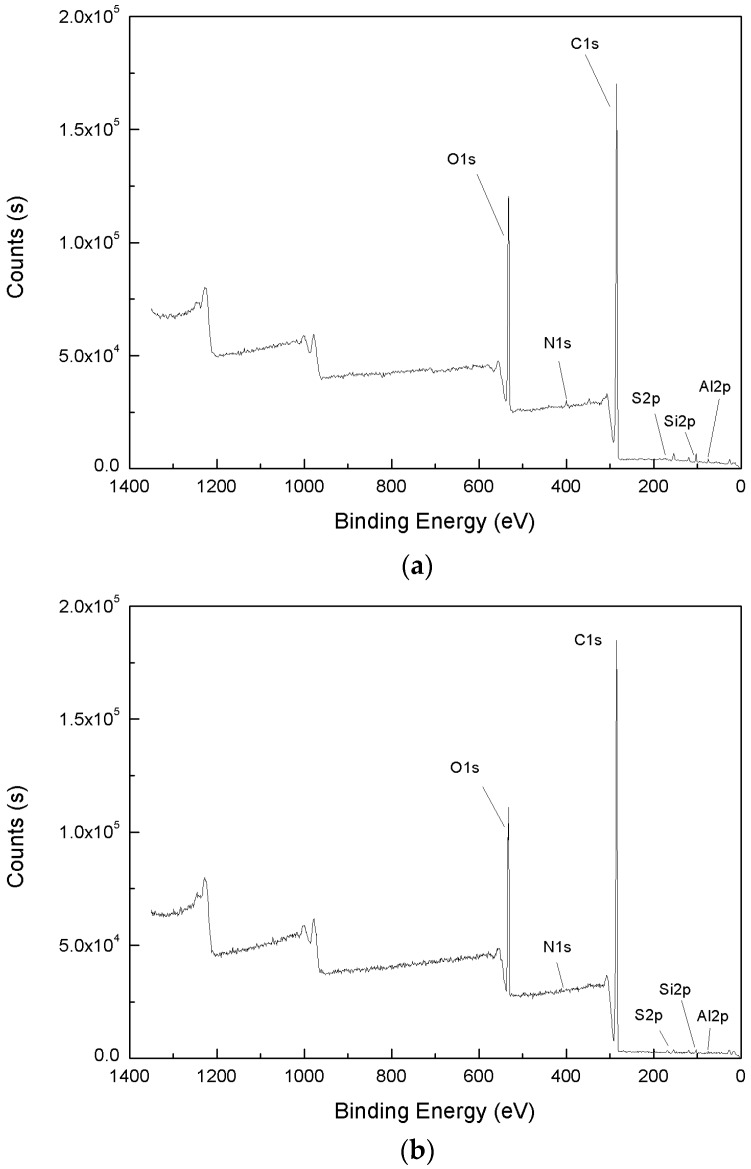
XPS survey scans of the coal surface before (**a**) and after (**b**) adsorption of NPEO_10_.

A wide-scan spectrum in the binding energy range 0–1400 eV was obtained to identify and quantitatively analyze the surface elements present [30]. A typical XPS wide-scan spectrum of demineralized lignite coal is presented in Figure 2.

Figure 2 shows that peaks of oxygen and carbon represent the major constituents of the coal surface both before and after the adsorption of NPEO_10_. We found clear changes in the O 1s, N 1s, C 1s, S 2p, Si 2p, and Al 2p peaks after adsorption of NPEO_10_. Compared with the case before NPEO_10_ adsorption, the C 1s peak is greatly enhanced after adsorption of NPEO_10_. However, the O 1s peak showed a clear reduction in size, and the N 1s, S 2p, Si 2p, and Al 2p peaks were slightly weakened. Quantitative peak analysis was carried out to determine the surface elements’ concentrations, and the results are shown in Table 2.

Table 2 shows that the contents (at %) of C 1s, O 1s, N 1s, S 2p, Si 2p, and Al 2p on the coal surface were, respectively, 76.58, 19.00, 1.12, 0.11, 1.82, and 1.37 at % before adsorption and 80.57, 17.33, 0.48, 0.25, 0.76, and 0.61 at % after adsorption. The content of C 1s increased from 76.58% to 80.57% after adsorption. However, the content of O 1s decreased from 19.00% to 17.33%. These results indicate that the oxygen functional groups on the lignite surface were significantly covered by NPEO_10_.

### 2.3. Molecular Dynamics Simulation of NPEO_10_ Adsorbed on the Coal Surface

#### 2.3.1. Structure of NPEO_10_ Adsorbed on the Coal Surface

**Figure 3:**
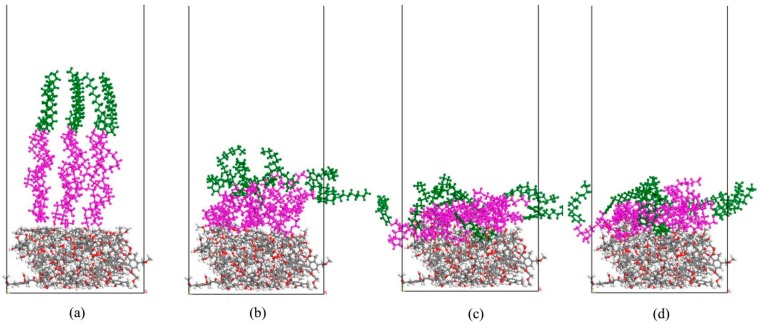
Simulation snapshot of NPEO_10_ (nonylphenol: green, and ethoxylate: purple) aggregates on lignite surface for different simulation times. The snapshots are taken at 0 ps, 250 ps, 500 ps, and 1 ns for (**a**–**d**) respectively (O: red, C: gray and H: white). For clarity, the water molecules are not shown.

**Figure 4:**
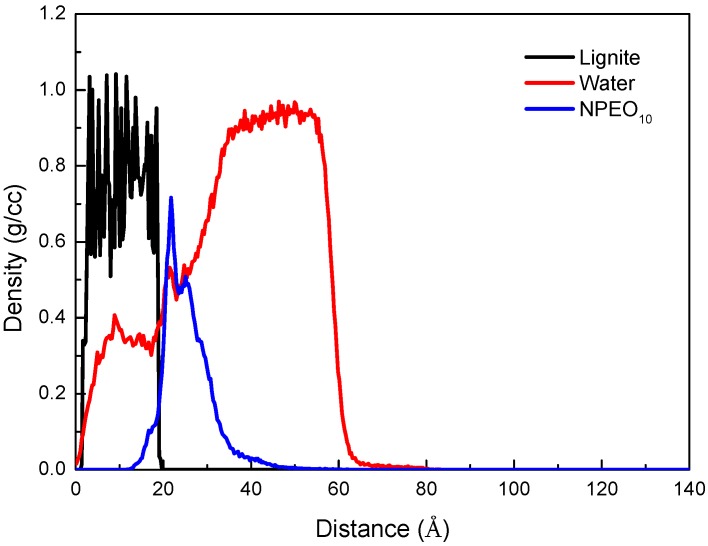
Density distributions of coal, NPEO_10_, and water molecules along the *Z* axis direction.

The aggregated structures of NPEO_10_ on a lignite surface for different simulation times are shown in Figure 3. The Z-dependent (Z being normal to the coal surface) density profiles for equilibrated configuration (1 ns) were calculated, and the density distributions of coal, NPEO_10_, and water molecules along the Z axis are shown in Figure 4.

The original configuration of NPEOs was in such a way with the polar head groups facing the lignite surface as can been seen in Figure 3a. As expected, after a short period of simulation, as shown in Figure 3b, due to the abundant hydrophilic oxygen functional groups of the lignite surface, the surfactant molecules try to reorient themselves so that their ethylene oxide groups adsorb lying on the coal surface through hydrogen bonds. Additional, the alkyl chains clearly highly intertwine with each other resulting from hydrophobic interaction. As time evolves, the hemimicelle structure forms on the lignite surface as, observed in Figure 3b,d. Figure 4 shows that the NPEO_10_ peak appears at ~20 Å, close to the first peak corresponding to water. This water layer may consist of the near-surface water film controlled by the hydrogen bonding between adsorbed water molecules and the coal surface. The intensity of the first peak of the water is obviously weaker, meaning there are fewer water molecules in this region. Most water molecules appear at a distance along the Z-axis exceeding 40 Å. The NPEO_10_ molecules exist at the interface between water and coal. Water molecules are repelled from the coal surface because of the stronger hydrophobicity of the lignite surface after adsorbing NPEO_10_. The results also show that the densities of NPEO_10_ and the coal surface overlap, which does not necessarily mean that NPEO_10_ molecules penetrate the lignite. Instead, the overlap is partly caused by the roughness of the surface, whose microscopic valleys are filled with the surfactant molecules.

**Figure 5:**
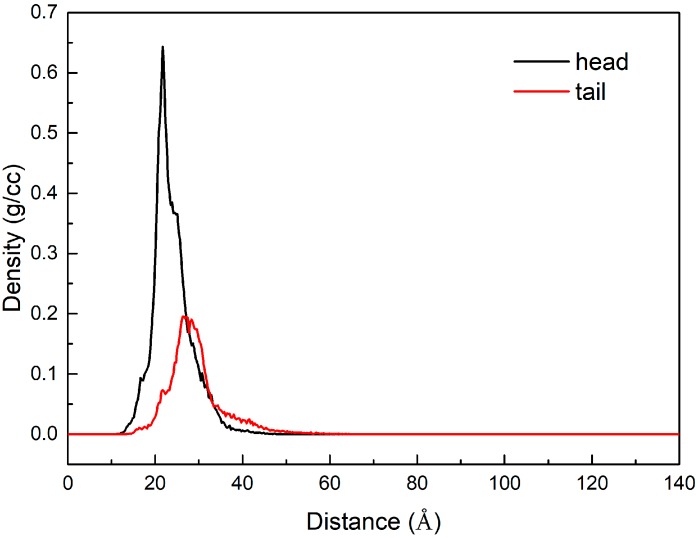
Density profiles of the NPEO_10_ head group and tail group along the Z direction.

The Z-dependent mass density profiles for the head (ethoxylate) and tail (nonylphenol) groups of NPEO_10_ were also calculated to survey the configuration of the adsorbed surfactant molecules on the coal surface, as shown in Figure 5. The peak of the head group was closer to the lignite surface than that of the tail group. Therefore, the non-ionic hydrophilic head groups are located next to the coal surface. As is well-known, the surface of coal is hydrophobic with hydrophilic sites, which means that there is an excess of head groups attached to the lignite surface. The polar interaction between the ethoxylated group of NPEO_10_ and the hydrophilic sites on the coal surface is the main force affecting the adsorption process. In other words, the ethylene oxide groups of NPEO_10_ preferentially adsorb on the hydrophilic sites of lignite and leave the hydrophobic portion of the molecule exposed to the solution. This result indicates that there is a high oxygen content and an abundance of hydrophilic surface functional groups on the lignite surface.

#### 2.3.2. Interaction Energies between Surfactant and Coal

The relative intensity and efficiency of the interaction between the surfactant and the coal surface is indicated by the interaction energy (E_*inter NPEO* & *coal*_). The value of E_*inter NPEO* & *coal*_ between NPEO_10_ and the lignite surface can be calculated using Equation (4):

(4) $$E_{inter\;NPEO\;\&\;coal}=(E_{total}-E_{NPEO}-E_{coal+water}-E_{coal}-E_{water+NPEO}+E_{water}+E_{NPEO+coal})/2$$

where E*_total_* is the total energy of the system. E*_coal_*, E*_NPEO_*, and E*_water_* refer to the energies of the coal surface, NPEO_10_, and water, respectively. E_*coal* + *water*_, E_*NPEO* + *water*_, and E_*coal* + *NPEO*_ are the total energy of coal and water, the total energy of NPEO_10_ and water, and the total energy of coal and NPEO_10_, respectively. The value of E_*inter NPEO* & *coal*_ obtained from the simulation is −174.16 kJ/mol. The negative value of the interaction energy between the surfactant and coal means that the system becomes more stable after adsorption.

#### 2.3.3. Mobility of Water Molecules

The mean square displacement (MSD) is the statistical average of particle trajectories, and is a measurement of the average distance of particles from a given particle. The dynamic properties of water molecules can also be obtained from the MSD, which can be expressed as follows [31]:

(5) $$\mathrm{MSD}=N^{-1}\langle \sum_{i}|r_{i}(t)-{r_{i}(0)|}^{2}\rangle$$

where *N* is the atom number, *r_i_*(0) represents the position vector at an initial time, and *r_i_*(*t*) represents the position vector after time t, and the angular brackets signify the ensemble average.

**Figure 6:**
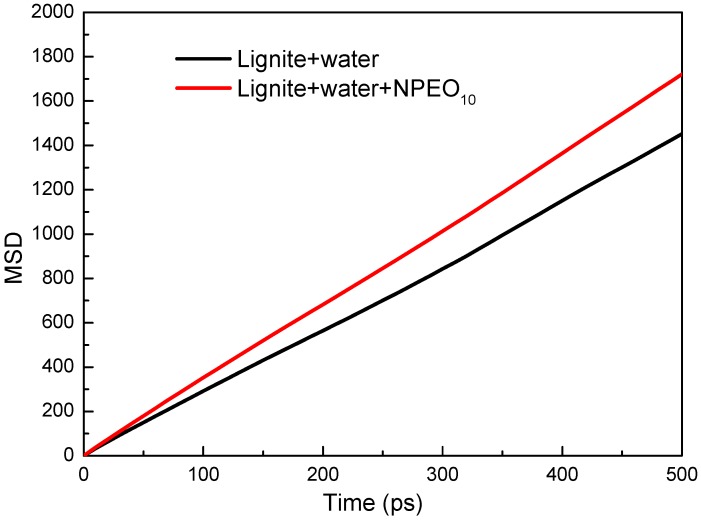
MSD curves of water molecules in the absence and presence of NPEO_10_; self-diffusion coefficients were calculated and assigned to their corresponding curves.

The MSD curves of water molecules in the absence and presence of NPEO_10_ are shown in Figure 6. It was evident that the mobility of water molecules was affected by the presence of NPEO_10_. Obviously, the increase in diffusion strength is greater in the coal-water-NPEO_10_ system.

The self-diffusion coefficient (D) reflects the intensity of atomic mobility of water molecules and was calculated in mixed systems both with and without NPEO_10_. D can be expressed as follows according to Einstein’s equation [32]:

(6) $$D=\frac{1}{6N}\underset{t\to \infty }{\lim}\frac{d}{dt}\sum_{i\to j}N\langle |r_{i}(t)-{r_{i}(0)|}^{2}\rangle$$

The MSD and self-diffusion coefficient are closely related, as follows:

(7) $$D=\lim\left(\frac{MSD}{6t}\right)=\frac{1}{6}K_{MSD}$$

The self-diffusion coefficients were calculated to be 5.67 × 10^−5^ and 4.79 × 10^−5^ cm^2^/s in the mixed system with and without NPEO_10_, respectively. This result means that the mobility of water over the modified coal surface caused by the adsorption of NPEO_10_ is enhanced as compared to that of water over the surface of original coal. The high mobility of water molecules should contribute to their displacement from the modified coal surface and the attachment of air bubbles. These simulated results are consistent with those obtained from XPS analysis.

## 3. Experiment and Methods

### 3.1. Materials

The lignite used in this study was provided by a colliery in China and was crushed to −0.5 mm. Analysis of the coal samples showed that its moisture content (*M*_ad_), ash content on a dry basis (*A*_ad_), volatile content (*V*_ad_), and fixed carbon content (*FC*_ad_) were 9.10%, 19.82%, 62.75%, and *FC*_daf_ = 37.25%, respectively.

**Figure 7:**
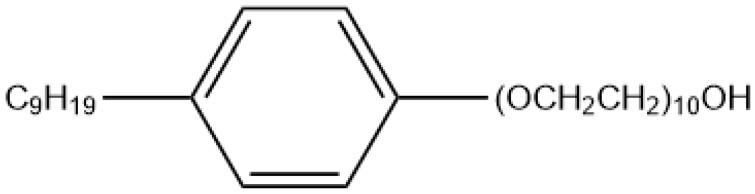
Structure of NPEO_10_.

NPEO_10_ was obtained from Union Carbide Chemicals (Danbury, Connecticut, USA) with no further purification. The chemical structure of NPEO_10_ is shown in Figure 7.

### 3.2. Surfactant Adsorption

The maximum adsorption density was measured for NPEO_10_ on the sample under investigation. The adsorption experiments were carried out in scintillation vials. Each vial contained surfactant solution (25 mL) of a known concentration and 0.5 g of the coal sample. The vials were agitated in a constant temperature water bath oscillator at 308, 318, and 328 K for 24 h, respectively, followed by suction filtration to obtain clear solutions. The surfactant concentrations in the solutions were determined using a spectrophotometer (Model UV757CRT) at a wavelength of 275 nm.

### 3.3. XPS Measurements

The XPS experiments are carried out at room temperature in an ultra-high vacuum (UHV) system with the surface analysis system (ESCALAB250 Xi, Thermo Fisher Scientific, Hudson, NH, USA). The spectrum of the survey scan is recorded at the pass energy of 100 eV with the step size of 1.00 eV. The high resolution spectra are recorded at the pass energy of 20 eV with the step of 0.05 eV. The data processing (peak fitting) is performed with the XPS peak fit software, using a Smart-type background subtraction and Gaussian/Lorentzian peak shapes.

### 3.4. Molecular Dynamics Simulation Methodology

**Figure 8:**
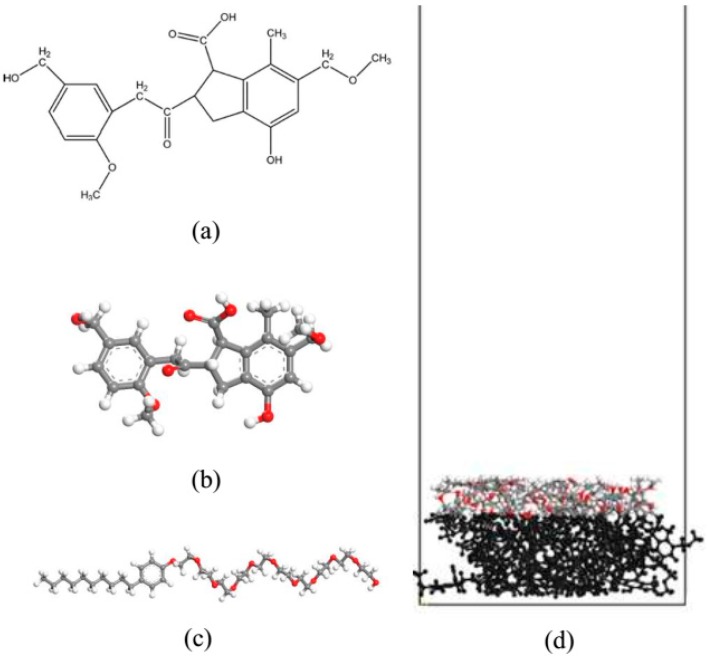
(**a**) Molecular model of lignite; (**b**) The structure of a single lignite model; (**c**) NPEO_10_ molecule model; (**d**) The structure of 20 optimized lignite models. The fixed atoms in the lignite model are shown in black. Colored balls represent O in red, C in gray, and H in white.

Molecular dynamics simulations were conducted using the Materials Studio 8.0 package. The COMPASS force field was applied in all simulations [33]. The molecular model of lignite was constructed based on Wender’s model [34] in combination with Kumagai’s model [35] and Tang’s model [36], as shown in Figure 8a. The structure of the lignite model was optimized as shown in Figure 8b. Then, 20 optimized lignite macromolecules were immersed in a periodic box. The system was equilibrated in a constant pressure-temperature (NPT) ensemble using a Berendsen thermostat and barostat. The pressure of the system was maintained at 0.1 MPa and the temperature was set to 298 K. A time step of 1.0 fs was used to integrate the equations of motion. A van der Waals interaction cutoff of 12.5 Å was employed, and the Ewald summation method was used to account for the long-range electrostatic interactions. After an initial equilibration for about 500 ps in an NPT ensemble, the lignite model was built with density of 1.22 g/cm^3^ as shown in Figure 8c. The structure of NPEO10 was selected for this study as shown in Figure 7 and the molecule models are shown in Figure 8d. The coal-water-NPEO system, which included 20 lignite macromolecules, nine NPEO10 molecules, and 2000 water molecules, was packed in a rectangular simulation cell 40 × 40 × 170 Å (X × Y × Z) with three-dimensional periodic boundary conditions. The simple point charge (SPC) water model [37] was used. The water potential parameters are listed in Table 3.

The molecular dynamics simulations were run at the NVT ensemble level at 298 K using a Nose thermostat, and the time step was set to 1.0 fs. A van der Waals interaction cutoff of 12.5 Å was employed, and the Ewald summation method with an accuracy of 10^−3^ kcal/mol was used to account for the long-range electrostatic interactions. The coal surface was frozen during the simulation to save computational effort, while the surfactant and water were allowed to relax. A simulation was performed for 1 ns. The final results were calculated based on the outcome of simulations of a period of 500 ps after the equilibration period.

**Figure 9:**
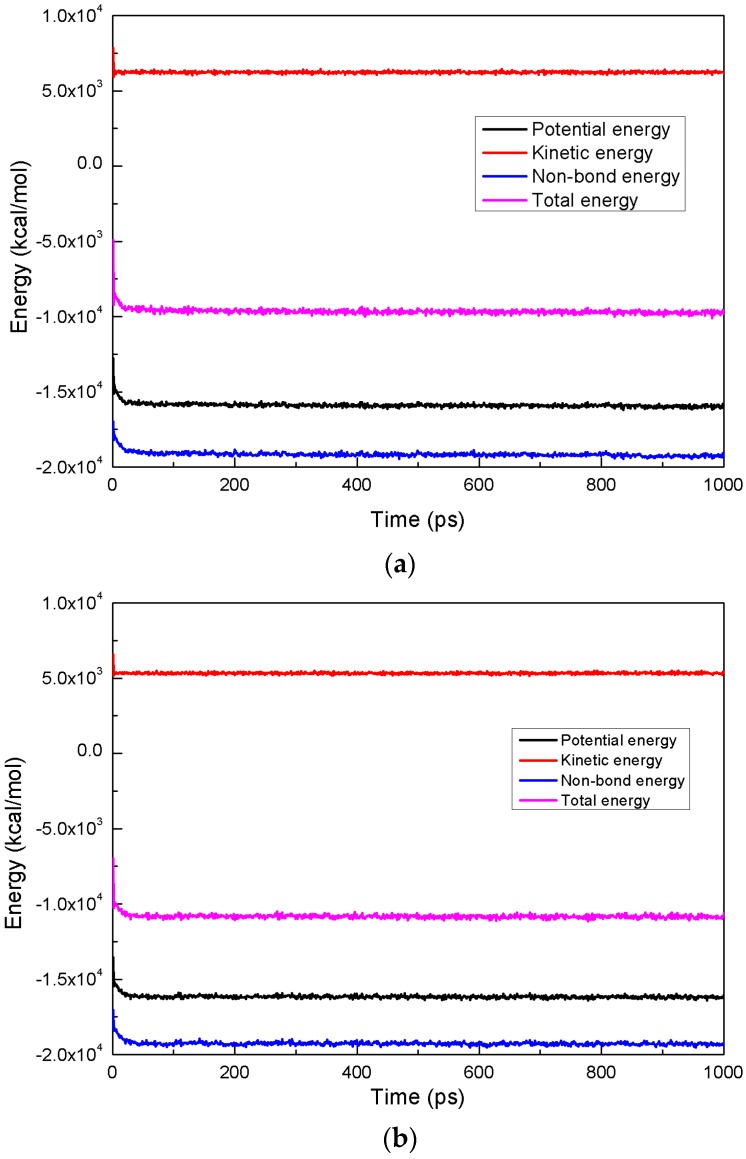
The fluctuation curves of energy during energy minimization. (**a**) Coal-water- NPEO_10_ system; and (**b**) coal-water system.

Curves showing the fluctuation of energy during the processes of energy minimization and annealing are shown in Figure 9. The potential energy, non-bond energy, kinetic energy, and total energy rapidly decreased to a minimum state and remained stable.

## 4. Conclusions

The adsorption behavior of nonylphenol ethoxylate with 10 ethylene oxide groups (NPEO_10_) on the surface of lignite was investigated by experimental and computational simulation methods.

The adsorption of NPEO_10_ on lignite follows the Langmuir-type isotherm at different temperatures. The thermodynamic properties of the adsorption process shows that the whole process is spontaneous and driven by both enthalpy and entropy synergistically. The X-ray photoelectron spectroscopic (XPS) analyses show that most of the oxygen-containing functional groups on the lignitic coal surface were covered by NPEO_10_.

Molecular dynamics (MD) simulations were used to investigate the adsorption behavior of NPEO_10_ on a model lignite surface. The NPEO_10_ molecules were found to adsorb at the water-coal interface. Moreover, the polar interactions between the ethoxylate group of NPEO_10_ and the hydrophilic sites on the lignitic coal surface were the main factor in the adsorption process. The density distributions of coal, NPEO_10_, and water molecules along the Z-axis direction showed that the remaining hydrophobic portions of surfactant, which extend into the solution, create a more hydrophobic coal surface to repel the water molecules.

The aggregated structure of adsorbed NPEO_10_ molecules was studied through the density profiles of the head and tail groups in the three spatial directions. The results showed that the negative interaction energy between the surfactant and the lignite surface suggest that the adsorption process is spontaneous, which is consistent with the thermodynamics of the experiment. The self-diffusion coefficients show that the presence of NPEO_10_ causes higher water mobility, improving the hydrophobicity of lignite.

## Figures and Tables

**Table 1:** Langmuir and Freundlich isotherm constants for NPEO_10_ onto coal sample coal at different temperatures.

*T*, *k*	Langmuir				Freundlich	
	*Q_m_*, mg g^–1^	*b*, L mg^–1^	*b*, L mol^–1^	*r*_1_^2^	*n*	*r*_2_^2^
308	41.67	60.00	3.96 × 10^7^	0.9990	0.12	0.5144
318	60.24	41.50	2.74 × 10^7^	0.9996	0.10	0.7136
328	81.30	30.75	2.03 × 10^7^	0.9992	0.09	0.6077

**Table 2:** Contents of C 1s, O 1s, N 1s, S 2p, Si 2p, and Al 2p on the coal surface before and after adsorption of NPEO_10._

Types	Before		After	
	Peak BE/eV	Contents/%	Peak BE/eV	Contents/%
C 1s	284.78	76.58	284.82	80.57
O 1s	532.47	19.00	532.79	17.33
N 1s	399.65	1.12	400.00	0.48
S 2p	163.30	0.11	168.12	0.25
Si 2p	103.36	1.82	103.14	0.76
Al 2p	74.86	1.37	74.73	0.61

**Table 3:** Water potential parameters.

*r*_OH_	*θ*_(HOH)_	*q*_(O)_	*q*_(H)_
0.1 nm	109°28′	−0.82 e	0.41 e
